# Involvement of Autonomic Nervous Activity Changes in Gastroesophageal Reflux in Neonates during Sleep and Wakefulness

**DOI:** 10.1371/journal.pone.0083464

**Published:** 2013-12-13

**Authors:** Djamal-Dine Djeddi, Guy Kongolo, Erwan Stéphan-Blanchard, Mohamed Ammari, André Léké, Stéphane Delanaud, Véronique Bach, Frederic Telliez

**Affiliations:** 1 PériTox Laboratory (EA4285-UMI 01 INERIS), Faculty of Medicine, Jules Verne University of Picardy, Amiens, France; 2 Pediatric Gastroenterology Unit, Amiens University Medical Center, Amiens, France; 3 Neonatal and Pediatric Intensive Care Unit, Amiens University Medical Center, Amiens, France; 4 GRAMFC (INSERM U 1105), Faculty of Medicine, Jules Verne University of Picardy, Amiens, France; University of Modena and Reggio Emilia, Italy

## Abstract

**Background:**

It has been suggested that disturbed activity of the autonomic nervous system is one of the factors involved in gastroesophageal reflux (GER) in adults. We sought to establish whether transient ANS dysfunction (as assessed by heart rate variability) is associated with the occurrence of GER events in neonates during sleep and wakefulness.

**Methods:**

Nineteen neonates with suspected GER underwent simultaneous, synchronized 12-hour polysomnography and esophageal multichannel impedance-pH monitoring. We compared changes in HRV parameters during three types of periods (*control* and *prior* to and *during* reflux) with respect to the vigilance state.

**Results:**

The vigilance state influenced the distribution of GER events (P<0.001), with 53.4% observed during wakefulness, 37.6% observed during active sleep and only 9% observed during quiet sleep. A significant increase in the sympathovagal ratio (+32%, P=0.013) was observed in the period immediately prior to reflux (due to a 15% reduction in parasympathetic activity (P=0.017)), relative to the control period. This phenomenon was observed during both wakefulness and active sleep.

**Conclusion:**

Our results showed that GER events were preceded by a vigilance-state-independent decrease in parasympathetic tone. This suggests that a pre-reflux change in ANS activity is one of the factors contributing to the mechanism of reflux in neonates.

## Introduction

Gastroesophageal reflux (GER) is defined as the physiologic passage of gastric contents into the esophagus. The phenomenon is very frequent in neonates, since it occurs in more than two-thirds of otherwise healthy infants [1-3] during the first months of life. These reflux events can have a strong impact on health because between 10% and 60% of infants show GER-related symptoms (such as bradycardia, apnea, faintness, etc.) [3].

Although GER has several causes, it has been shown that transient lower esophageal sphincter relaxation is a key factor in the occurrence of GER events in healthy preterm and term infants [4,5]. However, the mechanisms underlying the transient lower esophageal sphincter relaxation have not been well characterized.

In adults, GER has been linked to disturbances in autonomic nervous system (ANS) activity. This assumption is based on the observation of a higher prevalence of ANS disturbances in patients with GER disease. Given that vagal activity dysfunction is observed in both the presence and absence of inflammatory changes in the esophagus, some researchers have suggested that parasympathetic dysfunction is the prime factor in the etiology of GER and not just the consequence of esophageal inflammation [6,7]. It has further been hypothesized that disturbances in ANS activity could impair contraction of the lower esophageal sphincter (which normally acts as a reflux barrier) and may be involved in transient lower esophageal sphincter relaxation (and therefore also GER) [6,7]. Lastly, it has been suggested that ANS changes have a role in GER in neonates [8].

To the best of our knowledge, the temporal relationships between GER and ANS activity have not been studied in either adults or neonates. This is especially surprisingly in a neonatal context because the ANS is often still in the process of maturing during the first few months of life [9].

Therefore, the present study aimed at establishing whether GER events are associated with ANS activity changes in neonates. We particularly wanted to establish whether a putative disturbance of ANS activity occurred before and/or during GER. The GER events were investigated with multichannel intraluminal impedance-pH (MII-pH) monitoring [10]. Heart rate variability (HRV) analysis was used as a non-invasive method of assessing sympathetic-parasympathetic activities [11]. Vigilance states (sleep states and wakefulness (W)) were examined because of their well-known influence on gastrointestinal tract functions [12,13] and ANS activity [9].

## Materials and Methods

### Ethics statement

The study protocol had been approved by the regional investigational review board (*Comité de Protection des Personnes dans la Recherche Biomédicale de Picardie*). Before the start of the study, all parents had given their written, informed consent to their respective infant's participation.

### Patients

Combined MII-pH monitoring and polysomnography were performed in the Department of Pediatrics at Amiens University Medical Center (Amiens, France). The infants had been referred by their attending physician for investigation of GER symptoms (such as regurgitation, vomiting and/or belching at least 5 times per day for a week, associated with significant irritability, constituting warning signs for GER disease) [2]. The patients were enrolled consecutively from February 2007 to June 2008. Other inclusion criteria were a gestational age of 29-41 weeks and a post-conceptional age of 37-49 weeks. None of the infants had major gastrointestinal diseases, signs of esophagitis, congenital anomalies, neurological impairments or cardiorespiratory problems. None had been given drugs known to influence gastrointestinal motility or gastric pH. All the neonates were fed orally with their usual formula every 3–4 h.

### Protocol

All neonates underwent simultaneous, synchronized 12-hour polysomnography (PSG) and MII-pH monitoring. The MII-pH monitoring and PSG signals were recorded simultaneously on the polysomnograph and synchronized by using a SleepLab^®^ interface (Medical Measurement Systems BV, Enschede, The Netherlands). To avoid any bias due to potential circadian variations, all the recordings started at 07:00 PM on day 1 (after the evening feed) and continued until the final awakening at 07:00 AM on Day 2. The overall duration of each recording session was therefore about 12 h. The clothed neonates slept in the supine position in a crib located in a room that was isolated from routine nursing activities, noise and changes in light levels. 

### Multichannel intraluminal impedance and pH monitoring

All infants underwent MII-pH monitoring (with the Ohmega^®^ system from Medical Measurement Systems BV), in compliance with current European guidelines [14]. Multichannel intraluminal impedance recording is a procedure for measuring the movement of fluids, solids, and air in the esophagus. This technique provides a more detailed description of esophageal events and a more rapid response time than current pH-monitoring technology. The system included a portable data logger with impedance-pH ampliﬁers and a combined impedance-pH catheter with an outer diameter of 2 mm (Unisensor^®^, Attikon, Switzerland), containing one pH-measuring electrode and seven impedance sensors (at 2 cm intervals). Each pair of impedance electrodes corresponded to an impedance-measuring segment and thus a recording channel. The pH electrode was calibrated with standard buffers before the procedure. Each catheter was used once only and none malfunctioned during the investigations. Before the first feed on the day of the monitoring session, the MII-pH catheter was placed transnasally, positioned according to Strobel's formula [15] and then checked radiographically. The signals from the MII and pH channels were digitized at sampling rates of 50 Hz and 1 Hz, respectively. Moreover, MII-pH plots were analyzed automatically by using dedicated software (MMS Database^®^, version 8.9a, Medical Measurement Systems BV) and visually checked by a specialist in pediatric gastroenterology (DD). Feeding periods were excluded from the analysis. Based on the characteristics of the impedance signal, each GER event was deﬁned as liquid, gaseous or mixed. Retrograde liquid GER was deﬁned as a 50% decrease in impedance on at least two of the distal channels. Depending on the characteristics of the concomitant pH changes (as detected by the pH electrode), impedance-detected GER events were classiﬁed as acid reflux (pH<4, AR), weakly acid reflux (4<pH<7, WAR) or alkaline reﬂux (pH>7, AlkR). All reflux events with bolus movement detected by impedance were referred to as GER-imp events (i.e. liquid or mixed events). All reflux events detected by the pH electrode only (i.e. in the absence of bolus retrograde migration) were referred to as GER-pH events.

We considered all GER events in our analysis and did not analyze solely gaseous reflux. The following variables were analyzed: the total number and the frequency (h^-1^) of GER-pH and GER-imp events. The reflux index (RI) was defined as the percentage of time with pH<4 and the bolus exposure index (BEI) was defined as the time with GER-imp events as a percentage of total recording time [16].

### Polysomnography

The following signals were recorded continuously by PSG (the Brainnet-Morpheus system from Medical Data Technology^®^, Brussels, Belgium): (i) two electro-encephalograms from the right and left centro-occipital leads, (ii) two electro-oculograms, (iii) an electrocardiogram (ECG), (iv) chest and abdominal wall motion (with respiratory inductance plethysmography), (v) actimeter signals from a wrist and the opposite ankle and (vi) a synchronized, time-lapsed video signal (Handycam^®^, Sony, Tokyo, Japan) framing the infant’s face and body, in order to facilitate sleep scoring. Wakefulness and sleep stages (active sleep: AS; quiet sleep: QS) were scored visually in 30-second epochs, as recommended by Anders et al. [17]. Active sleep was defined as continuous EEG activity with rapid eye movements, whereas QS was defined as discontinuous EEG activity in the absence of rapid eye movements. Wakefulness was defined as a state in which the infant’s eyes were open and body movements were frequent. Periods that did not fulfill the criteria for either AS or QS were scored as indeterminate sleep.

### Heart rate variability analysis

The ECG was recorded via three-lead electrodes placed on the precordial area. The ECG signal was digitized at a sampling rate of 400 Hz. Data were analyzed using a peak detection algorithm that identified the R wave of the QRS complexes after all motion artifacts had been edited out. A QRS detection algorithm was implemented, in order to locate a stable, noise-independent fiducial point on the R wave. By comparison with the adjacent morphologic features, the QRS complexes were automatically classified (and then visually checked) as normal sinus rhythm, atrial or ventricular premature beats or noise. The normal-to-normal RR intervals (NN intervals) were deduced from adjacent normal sinus beats. Each ectopic beat (i.e. a beat that differed by at least two SD from the mean RR interval) was identified and replaced by the mean RR value of the three-minute segment studied. Each three-minute segment of NN intervals was extracted for HRV analysis and aligned with a period of MII-pH data. A beat-to-beat HRV signal was then computed. The data were re-sampled at 10 Hz (using linear interpolation) to obtain an equally sampled time series. A continuous function was generated from the sequence of NN intervals recorded on the ECG. Standardized time- and frequency-domain HRV analyses were performed on successive three-minute segments of data recorded throughout the night [18].

#### Time-domain analysis

The following parameters were calculated: the SD of the differences between consecutive NN intervals (SDSD in sec, which describes short-term variations in the heart rate), the square root of the mean of the squared differences between adjacent NN intervals (RMSSD in sec, which describes high-frequency variability) and the proportion of adjacent NN interval differences that were >50 msec (pNN50 in %). Each of these parameters reflects parasympathetic activity.

#### Frequency-domain analysis

A fast Fourier transform was used to convert time series into the frequency domain. The direct current component was deleted and a Hamming window was applied. For each time segment, the power spectrum densities were estimated using Welch’s averaged periodogram method [19]. Our spectral analysis of HRV focused on (i) the very low-frequency component (VLF, 0.003-0.04 Hz), the physiological significance of which remains unclear but may be related to the thermoregulatory and renin-angiotensin systems, (ii) the low-frequency component (LF, 0.04-0.15 Hz), which reflects sympathetic and parasympathetic tones, and (iii) the high-frequency component (HF, 0.15-0.4 Hz), which reflects parasympathetic activity. The total variability (VLF+LF+HF) represents the total power in the spectrum for the region analyzed. The LF and HF powers were expressed as absolute values and were also converted into normalized units (LF_nu_, HF_nu_) by dividing the period's power by the total spectral power minus the VLF (i.e. LF+HF). Sympathovagal balance was expressed as the LF/HF ratio.

### Data analysis

All GER-imp and GER-pH events were considered in our analysis of the temporal relationship between ANS activity and reflux event occurrence. To assess the changes in ANS activity in relation to GER, we examined the changes in HRV parameters during three periods of three minutes defined with regard to each GER event's time of occurrence: (i) a *control* period (free of reflux events), (ii) a period immediately *prior to* the GER and (iii) a period *during* the GER. The latter period started at the beginning of the GER (as determined by a pH<4 and/or a drop of 50% in impedance on at least 2 of the distal channels). The three-minute *control* period was located immediately before the *prior* period. 

To be included in our analysis, a GER event had to meet several methodological criteria: the three periods had to be (i) located within the same vigilance state and (ii) free of body movements and/or apnea, so that the ECG signal was not disrupted. When several reflux events occur in successive three-min periods, we only considered the first reflux event in our analysis.

### Statistical analysis

Statistical analyses were performed with Statview software (version 5.0, SAS Institute Inc., Cary, NC). 

A χ^2^ test was used to test for an influence of the vigilance state on the distribution of GER events. For each HRV parameter, a two-way analysis of variance for repeated measures and pairwise multiple comparison procedures were used to test the differences between the *control*, *prior* and *during* periods as a function of the vigilance state. Data were adjusted for multiple testing with the Tukey-Kramer test. *P*<0.05 was considered to be statistically significant. Test results with *P*<0.10 are also given, when relevant.

## Results

Nineteen neonates (mean ± standard deviation (SD) gestational age: 36.6 ± 3.5 weeks; birth weight: 2.6 ± 0.7 kg; post-menstrual age: 42.0 ± 4.9 weeks, body weight at the time of the study: 3.4 ± 1.2 kg) hospitalized for suspected GER were enrolled in this study. Seven of the infants were born preterm and the other twelve were born at term.

Esophageal MII-pH monitoring and sleep data are shown in Tables 1 and 2, respectively. Only five neonates had abnormal pH monitoring data (RI>7%; the abnormal RI sub-group) [2], with no significant differences in the other MII-pH parameters vs. the infants with normal pH monitoring data. We observed that 78.7% of the impedance-detected reflux events were weakly acid, 18.7% corresponded to acidic reflux and 2.6% corresponded to alkaline reflux (Table 3). 

**Table 1:** GER data detected by MII-pH monitoring for the 19 neonates.

Recording time, min	760 ± 130
**GER-pH events**	
RI (pH-monitoring), % of total time	4 ± 4
Mean number of GER-pH events, n	14 ± 15
GER-pH frequency, h^-1^	1.3 ± 1.7
**GER-imp events**	
BEI, % of total time	1.0 ± 0.7
Mean number of GER-imp events, n	41 ± 25
GER-imp frequency, h^-1^	3.6 ± 2.1

Values are quoted as the mean ± SD. RI: reflux index, BEI: bolus exposure index.

**Table 2:** Sleep data for the 19 neonates.

**Sleep parameters**	
Sleep period time, min	728 ± 114
Wakefulness duration, min.	180 ± 78
Total sleep time, min.	535 ± 82
**Sleep structure**	
Active sleep, %	61 ± 11
Indeterminate sleep, %	8 ± 5
Quiet sleep, %	31 ± 9
Frequency of sleep state changes, min^-1^	0.10 ± 0.03

Values are quoted as the mean ± SD.

**Table 3:** Reflux data as a function of vigilance state for the 19 neonates (χ^2^ test was used to test for an influence of the vigilance state on the distribution of GER events).

	**Wakefulness**	**Active sleep**	**Quiet sleep**	**Total**	**Vigilance state effect**
Total duration, min	2886	5237	2637	10760	P=0.0003
GER-pH events, n	23	43	39	105	P<0.05
GER-imp events, n	358	226	26	610	P<0.001
acid GER-imp	59	53	2		P<0.001
weakly acid GER-imp	285	172	24		P<0.001
alkaline GER-imp	14	1	0		P<0.001
Total GER events, n (%)	381 (53.4%)	269 (37.6%)	65 (9%)	715	P<0.001
GER frequency (h^-1^)	7.9	3.1	1.5		P<0.001
GER events considered in the HRV analysis	196	131	13	340	

### The influence of vigilance state

Vigilance state had an influence on the frequencies and distributions of both GER-pH and GER–imp events (Table 3). The GER frequency was lower in AS than in W and was lowest in QS. 91% of the GER events occurred in W and AS and so only 9% were observed in QS.

Following application of our methodological criteria, 340 of the 715 observed GER events were used in the HRV analysis. In the 19 neonates, valid HRV data (i.e. artifact-free ECGs during the *control*, *prior* and *during* periods in the same vigilance state episode) were available for 196 GER events in W (i.e. 51.4% of the GER during W) and 131 GER events in AS (i.e. 48.7% of the GER during AS). Given the low number of GER events during QS (n=13), we did not consider this sleep state in our analysis.

As often described in the literature [9], vigilance state had an effect on all the HRV time- and frequency-domain parameters (Tables 4 and 5, respectively). The mean duration of RR intervals was lower during W than during AS, although this difference was only indicative (*P*<0.10). The values for RMSSD, SDSD and pNN50 were significantly higher during W than during AS. Total variability and the absolute values of HF and LF were also higher during W than during AS. When LF and HF were expressed in normalized units, LF_nu_ was significantly lower during W than during AS, whereas HF_nu_ was significantly higher during W than during AS. As a result, the LF/HF ratio was significantly lower during W than during AS.

**Table 4:** Time-domain HRV parameter values during W and AS in three periods: a GER-free period (*control*), a period immediately prior to GER (*prior*) and a period during GER (*during*) (a two-way analysis of variance for repeated measures were used to test the differences between the *control*, *prior* and *during* periods as a function of the vigilance state).

	**W**			**AS**			**Vigilance state effect**	**Period effect**
**Period**	***control***	***prior***	***during***	***control***	***prior***	***during***	***P***	
**RR , s**	0.415±0.059	0.415±0.056	0.409±0.050	0.417±0.061	0.425±0.065	0.420±0.059	0.060	NS
**SDSD, s**	0.071±0.045	0.060±0.042	0.076±0.052	0.057±0.039	0.052±0.034	0.059±0.039	<0.001	0.022
**RMSSD, s**	0.011±0.016	0.008±0.016	0.013±0.021	0.006±0.012	0.005±0.009	0.007±0.016	0.001	0.038
**pNN50, %**	6.2±7.9	4.4±5.7	5.6±7.0	4.0±6.6	3.7±8.0	4.6±9.0	0.019	NS

Data were adjusted for multiple testing with the Tukey-Kramer test.

Values are quoted as the mean ± SD.

**Table 5:** Frequency-domain HRV parameter values during W and AS in three periods: a GER-free period (*control*), a period immediately prior to GER (*prior*) and a period during GER (*during*) (a two-way analysis of variance for repeated measures were used to test the differences between the *control*, *prior* and *during* periods as a function of the vigilance state).

	**W**			**AS**			**Vigilance state effect**	**Period effect**
**Period**	***control***	***prior***	***during***	***control***	***prior***	***during***	***P***	
**Total V, s^2^**	0.069±0.069	0.055±0.058	0.074±0.068	0.054±0.061	0.050±0.052	0.056±0.059	0.008	0.060
**LF, s^2^**	0.026±0.024	0.021±0.020	0.027±0.023	0.021±0.021	0.020±0.020	0.022±0.022	0.016	NS
**HF, s^2^**	0.028±0.038	0.018±0.031	0.028±0.037	0019±0.031	0014±0.023	0016±0.026	<0.001	0.025
**LF_nu_**	62 ±19	68 ±21	63 ±20	65±20	70±19	68 ±20	0.030	0.024
**HF_nu_**	38 ±19	32 ±21	37 ±20	35±20	30±19	32 ±20	0.030	0.024
**LF/HF**	3.2±3.5	4.2±4.3	3.3±3.5	3.7±4.4	4.9±5.3	4.9±6.5	0.007	0.045

Data were adjusted for multiple testing with the Tukey-Kramer test.

Values are quoted as the mean ± SD.

No interaction between vigilance states and the period (*control, prior* and *during GER*) was observed for any of the time- and frequency-domain HRV parameters. Hence, vigilance states were pooled in the subsequent analysis.

### Reflux-related changes in HRV

Of the 340 GER events analyzed in this study, 38 (11%) were GER-pH only events, 248 (73%) were weakly acid GER-imp events and 54 (16%) were acid GER-imp events. The mean duration was 13 ± 13 sec for a GER-imp event and 76 ± 62 sec. for a GER-pH event.

A significant influence of the period was found on the time-domain HRV parameters SDSD and RMSSD (Table 4). These parameters were significantly lower in the *prior* period than in the *control* period (SDSD: *P*=0.097 and RMSSD: *P*=0.064) and the *during* period (SDSD: *P*=0.006 and RMSSD: *P*=0.003). No influence of the period was observed for RR and pNN50.

**Figure 1:**
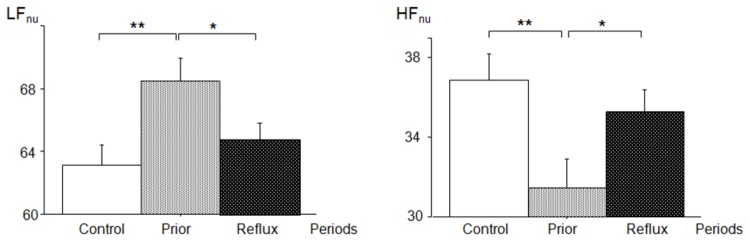
Effect of the period on normalized values (mean ± SEM) of the power spectra in the low- (LF_nu_) and high-frequency (HF_nu_) bands. *P<0.05, **P<0.01. (pairwise multiple comparison procedures were used to test the differences between the control, prior and during periods. Data were adjusted for multiple testing with the Tukey-Kramer test).

**Figure 2:**
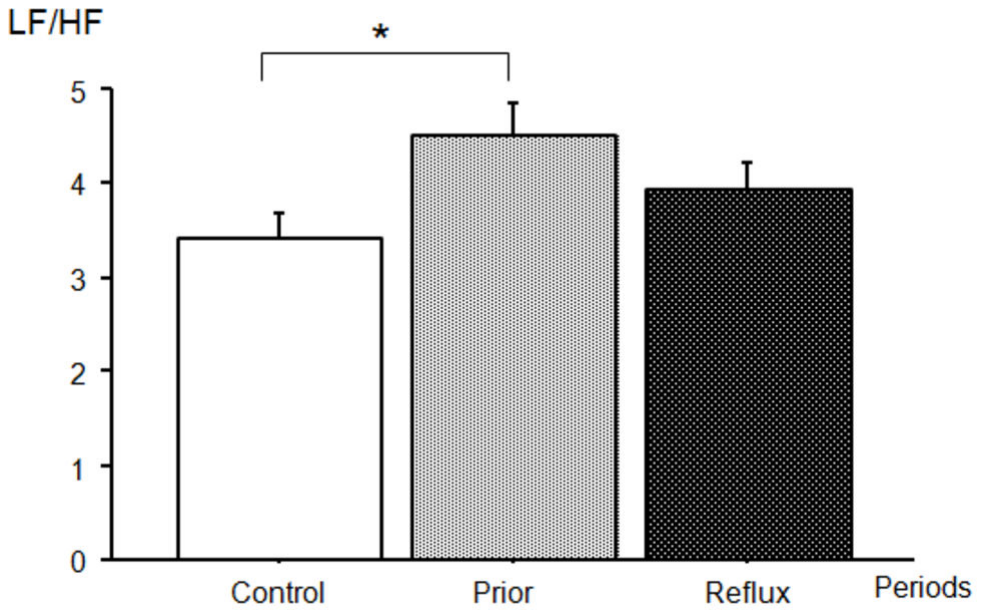
Effect of the period on the sympathovagal balance (LF/HF) (mean ± SEM). *P<0.05. (pairwise multiple comparison procedures were used to test the differences between the *control*, *prior* and *during* periods. Data were adjusted for multiple testing with the Tukey-Kramer test).

In the frequency domain (Table 5), a noteworthy period effect was observed for the absolute HF values. In the *prior* period, the HF power (0.017 ± 0.029 s^2^) was significantly lower than in the *control* period (0.024 ± 0.036 s^2^, *P*=0.013) and the *during* period (0.023 ± 0.033 s^2^, *P*=0.019). No significant inter-period difference was observed for absolute values of LF (*control*: 0.024 ± 0.023 s^2^, *prior*: 0.021 ± 0.020 s^2^, *during*: 0.025 ± 0.023 s^2^). When compared with the *control* period, HF_nu_ was 15% lower in the *prior* period (*control*: 36.9 ± 19.7%, *prior*: 31.5 ± 20.3%, *P*=0.005), whereas LF_nu_ was 8.5% higher (*control*: 63.1 ± 19.5%, *prior*: 68.5 ± 20.3%, *P*=0.005) (Figure 1). As a result, the LF/HF ratio was 32% higher in the *prior* periods (4.5 ± 4.7) than in the *control* periods (3.4 ± 3.9, *P*=0.030) (Figure 2).

Overall, 65% of the 340 GER episodes were associated with a decrease in HF_nu_ and an increase in the LF/HF ratio in the *prior* period. A decrease in parasympathetic activity and an increase in the sympathovagal balance in the *prior* period were observed in 16 of the 19 neonates (84%). 

Over the course of the *during* period, LF_nu_, HF_nu_ (Figure 1) and LF/HF (Figure 2) tended to return to their *control* values. The differences in these parameters with respect to the *prior* period were only significant for LF_nu_ (*P*=0.033) and HF_nu_ (*P*=0.034).

### Reflux-related changes in HRV: influence of GER-pH vs. GER-imp events, *abnormal RI* vs. healthy infant sub-groups, and term vs. preterm infants

We did not observe any influence of the type of reflux (GER-pH *vs.* GER–imp), acidity (acid GER–imp *vs.* weakly acid GER–imp) or mean duration of the GER event on our HRV results; the effect of the period on HF_nu_, LF_nu_ and LF/HF ratio did not appear to depend on the characteristics of the reflux.

The decrease in HF_nu_ (and increase in LF/HF) in the *prior* period was observed in infants born at term and infants born preterm. The decrease in HF_nu_ (and increase in the LF/HF ratio) immediately *prior* to reflux was observed in 4 out of 5 of the infants with RI>7% and 11 out of 14 of the healthy infants.

## Discussion

Gastroesophageal reflux has a marked impact on infant health and is potentially harmful in the first few weeks of life. Although a change in ANS activity is thought to be one of the factors involved in the occurrence of GER via transient lower esophageal sphincter relaxation, no direct evidence has been provided to date. To the best of knowledge, the present study is the first to have demonstrated (by using HRV analysis) that in neonates, GER may be preceded by significant changes in ANS activity (and primarily a decrease in parasympathetic activity).

### Characteristics of GER events

The frequency of GER events observed in the present study was quite similar to that reported by van Wijk et al. [20] in neonates with frequent regurgitation as detected by MII-pH monitoring (between 1.6 and 6 GER events per hour). The RI recorded in the present study (4%) is lower than the "abnormal" threshold of 7%^2^. In the literature, the correlation between abnormal esophageal pH events and symptoms appears to be especially strong when RI>10%^2^. Moreover in symptomatic, premature infants, Corvaglia et al. [21] found a high BEI (1.31%, *vs.* 1.0% in our study) and a higher RI (8.35%, *vs.* 4.0% in our study). Accordingly, we consider that our results are valid for this population but would have to be specifically confirmed in a population in which all newborns have GER disease.

#### The influence of the vigilance state

The vigilance state influenced the occurrence of GER-pH and GER-imp events. Reflux events were mainly observed during W and AS and more rarely during QS. Indeed, W accounted for 27% of the recording time and 53.4% of the GER events. During sleep, 37.6% of the GER events occurred during AS and only 9% occurred during QS. This result is in line with the literature data [12,22]. In the study of five-month-old infants by Machado et al. [22], the GER frequency was higher during W (4.3 GER events.h^-1^) than during sleep (2.6 GER events.h^-1^).

Our HRV analysis also showed that vigilance states did not influence the patterns of GER-related ANS activity. The changes in HRV parameters observed immediately prior to GER were similar in AS and in W. This result must be considered with a degree of caution, since our analysis was performed on only 53.4% and 47.8% of the GER events in W and AS, respectively. This methodological limitation was due to the need for three artifact-free, three-minute periods of HRV data (i.e. the absence of body movements and apnea) in the same vigilance state episode for each GER, which necessarily restricted the amount of analyzable data. Indeed, this meant that we could not take QS into account in the HRV analysis because of the low number of GER events in this sleep stage. In addition to methodological considerations, it is well known that QS is characterized by lower HRV, higher sympathovagal balance and a higher parasympathetic level [9] than in AS and W. This could partly explain the lower number of GER events found in our study and also argues in favor of the involvement of a transient decrease in parasympathetic activity in the occurrence of GER.

### Gastroesophageal reflux events and ANS changes

As mentioned above, nearly 50% of the GER events detected by MII-pH monitoring were not considered in the HRV analysis because the period prior to reflux was disrupted by body movements and/or apnea. This observation is in line with data literature data highlighting a strong association between GER and body movements [12,23]. The increase in abdominal pressure that results from body movement may temporarily overwhelm the pressure barrier at the base of the esophagus and may thus promote GER. Apnea may also be involved in GER [24]. Respiratory patterns and esophageal function (including that of the lower esophageal sphincter) are regulated by central pathways. A defect of impairment in central neural outputs may allow reflux and apnea to occur simultaneously [25]. This may be particularly important during the first few weeks of life (when the ANS may not have matured). In a context of obstructive apnea, the relationship between reflux and apnea may be due to a physical mechanism whereby negative intra-thoracic pressure sucks gastric content into the esophagus [26]. Furthermore, our present results do not rule out the involvement of other peripheral, physiologic triggers for GER, such as gastric distension (mediated by the vagovagal pathway and initiated by tension receptors located in the proximal stomach musculature) and abdominal straining – both of which are thought to increase the likelihood of GER events.

Under physiological conditions, normal esophageal reflux fluctuates on a time scale of a few seconds up until one or two minutes. However, a stable, reproducibility HRV analysis requires at least three minutes of recording. We may therefore have lost some statistical information by smoothing out changes in ANS activity during our HRV analysis. Nevertheless, our results clearly showed that a transient change in ANS activity (as measured in an HRV analysis) occurs immediately before the GER event. This change was mainly due to a decrease in parasympathetic activity (as evidenced by the decrease in SDSD, RMSSD, HF and HF_nu_). The present results support and extend the Tirosh et al.'s hypothesis [8] whereby autonomic alterations are associated with GER. The latter authors have shown that infants with a history of idiopathic, apparently life-threatening events display a significant HRV increase in the period preceding obstructive apnea. In contrast, no such change was found following a coupled apnea-GER event (as determined by pH-metry). Tirosh et al. concluded that a decrease in parasympathetic function was a specific mechanism related to GER.

Our present observations are in line with studies of adults [6,7] in which a role for the parasympathetic system in reflux disease has been suggested. The pressure exerted by the lower esophageal sphincter (as a barrier to reflux) is under the control of the ANS. In general, the lower esophageal sphincter must relax completely before GER can occur. The most common form of the lower esophageal sphincter relaxation associated with GER triggering is transient lower esophageal sphincter; this appears to be the predominant mechanism in GER and reportedly triggered 82% [4] of the GER events in healthy premature infants and 91.5% [5] of the GER events in preterm and term infants with GER disease. Disturbances in ANS activity (such as decreased vagal activity) could reduce myogenic control of the lower esophageal sphincter, favor the lower esophageal sphincter relaxation and thus probably increase the frequency of transient the lower esophageal sphincter relaxation. Moreover, anticholinergic drugs (such as atropine) decrease the lower esophageal sphincter pressure in infants [27] and adults [28], suggesting that a decrease in parasympathetic tone could be involved in the occurrence of GER [29]. However, the role of the parasympathetic system in lower esophageal sphincter relaxation is still subject to debate, since the neurotransmitters involved have not been fully identified. Acetylcholine, tachykinins, substance P and neurokinins A and B are the main excitatory neurotransmitters. Other neurotransmitters (such as nitric oxide) have a major role in relaxation. Hence, in theory, the decrease in parasympathetic activity could either increase or decrease the occurrence of transient lower esophageal relaxations. Parasympathetic tone (as assessed by HRV analysis) is an overall evaluation and may concern the activating and/or inhibitory pathways – it is not yet known. When a reflux event (and therefore lower esophageal sphincter relaxation) occurs, our results may suggest that a decrease in parasympathetic tone reflects depression of the vagal efferents of activating pathways controlling the cholinergic myenteric neurons that innervate the lower esophageal sphincter.

Here, HRV parameters tended to return to their control values during GER events, with an increase in HF_nu_ and a decrease LF_nu_ (when compared with the period *prior* to reflux). As a consequence, we did not find any difference between the control period and the period during GER. Thus, we did not see a significant impact of GER on cardiovascular reactivity *per se*. These results are not so different from those already reported in preclinical and clinical studies of adults. The esophagus has parasympathetic and sympathetic sensory innervations that are sensitive to both chemical and mechanical stimuli [30]. Artificial electrical, mechanical or chemical stimulation of the esophagus has been found to increase the vagal modulation of cardiac function [31]. Indeed, esophageal afferent stimulation can increase the HF vagal power [32]. However, the use of artificial stimulation to assess the impact of sensory visceral afferences on cardiac autonomic control in infants remains questionable from a physiological standpoint. It is also possible that arousal induced by GER (observed in 49.8% [22] and 73.5% [33] of GER), with increases in EMG activity and heart rate, may counteract this effect. Further analyses are required to discriminate cardiac impact of GER as regards to arousal response.

### Influence of term vs. preterm delivery, the type of reflux, and abnormal RI vs. normal RI

The temporal relationship between changes in autonomic nervous system activity and GER occurrence did not appear to be influenced by the type of reflux, the severity of the GER profile or term vs. preterm delivery. Our results agree with the report by Omari et al. [4,5], who found that transient lower esophageal sphincter relaxation is the main mechanism underlying GER when comparing pathologic *vs.* healthy infants and term vs. preterm infants. This may suggest that changes in parasympathetic activity are involved in the triggering of transient lower esophageal sphincter relaxations. To reinforce and extend our results, it would be interesting to use a neonate-specific manometry system to measure transient lower esophageal sphincter relaxations associated with reflux events and the latter's correlation with HRV parameters.

## Conclusion

The development of novel therapeutic strategies and medications for effective anti-reflux therapy in infants requires a better understanding of the mechanism that underlies GER. Our present results show that changes in the activity of the ANS (and mainly a decrease in parasympathetic activity) may contribute (at least to some extent) to the occurrence of GER. 
